# Food insecurity and gender-based violence against women during the COVID-19 pandemic: a systematic review

**DOI:** 10.1186/s12889-026-26253-3

**Published:** 2026-01-27

**Authors:** Hanan A. Abusbaitan, Kaboni W. Gondwe, Anna Pirsch, Anwar Eyadat, Nadeen Sami Alshakhshir, Nokuthula Vilakazi, Yamikani Nkhoma-Mussa, Mary O. Hearst, Lucy Mkandawire-Valhmu, Alexa A. Lopez, Diane M. Schadewald, Anne Dressel

**Affiliations:** 1 School of Nursing, Oakland University, Human Health Building 2036, 433 Meadow Brook Rd, Rochester, MI 48309-4452 USA; 2 School of Nursing, Department of Child, Family, and Population Health Nursing, University of Washington, Seattle, WA USA; 3 Nursing Program, Augsburg University, Minneapolis, MN USA; 4 Departmet of Community and Mental Health, Faculty of Nursing, The Hashemite University, Zarqa, Jordan; 5 School of Nursing, University of Minnesota, Minneapolis, MN USA; 6 Department of Consumer Sciences, Food and Nutrition, Durban University of Technology, Durban, South Africa; 7 School of Nursing, University of Wisconsin-Madison, Madison, WI USA; 8 School of Nursing, University of Wisconsin-Milwaukee, Milwaukee, WI USA

**Keywords:** Food insecurity, Gender-based violence, COVID-19, Social-ecological model, Intersectionality framework

## Abstract

**Background:**

Gender-based violence (GBV) and food insecurity are conditions that affect women and may also exacerbate each other, as women experience disproportionately higher levels of both globally. Both of these conditions also increased during the COVID-19 pandemic. The purpose of this systematic review was to describe how the association between food insecurity and GBV across multiple global regions during the COVID-19 pandemic impacted women. The review aims to inform equity-driven interventions and policy development in the event for use during future emergency crises.

**Methods:**

The social-ecological model (SEM) and the intersectionality framework were the frameworks used for this systematic review. The PRISMA guidelines guided this systematic review methodology. The literature search was conducted using the APA PsycINFO, CINAHL, MEDLINE, PubMed, and Web of Science databases in November 2025. The inclusion criteria were as follows: (1) studies conducted during the COVID-19 pandemic; (2) studies that assessed the association between food insecurity and GBV among women during the COVID-19 pandemic; and (3) studies published in English in peer-reviewed journals. The exclusion criterion was any study that was not primary research. The Quality Assessment Tool for Studies with Diverse Designs (QATSDD) was used for quality appraisal. Thematic analysis, guided by Hennink and colleagues (2020), was used to synthesize the results.

**Results:**

Thirty-two studies were included in the data analysis. At the individual level, food insecurity and GBV during the COVID-19 pandemic were associated with a greater likelihood of reporting mental health conditions such as anxiety and depression. Additionally, being an immigrant was associated with a high risk of experiencing food insecurity and GBV. At the relationship level, food insecurity and GBV were associated with household instability and family dysfunction. At the community level, the association was influenced by poverty and limited employment opportunities. At the societal level, restrictive COVID-19 policies and prevailing cultural norms contributed to intensifying food insecurity and GBV.

**Conclusion:**

This study offers support for strengthening crisis‒response systems across socio-ecological levels that incorporate gender-sensitive food security and violence prevention strategies during public health emergencies. New policies are needed to create effective support systems to promote women's health, especially marginalized groups, who experience the greatest vulnerability.

**Supplementary Information:**

The online version contains supplementary material available at 10.1186/s12889-026-26253-3.

## Background

Food insecurity and gender-based violence (GBV), two critical determinants of health, are significant concerns for social and public health professionals worldwide. GBV involves physical, mental, sexual, and economic acts of violence toward women that occur in private or public spaces. GBV encompasses intimate partner violence (IPV), domestic violence, child marriage, and female genital mutilation [1]. IPV primarily includes violence occurring in romantic relationships inflicted by husbands or intimate partners [2]. This systematic review focuses on both domestic violence and IPV affecting women during the COVID-19 pandemic. The information in the results section regarding poverty, financial distress, and related factors presented in relation to how these stressors were compounded by COVID-19 and contributed to food insecurity and GBV during the pandemic was also part of this analysis.

Addressing food insecurity and GBV is essential and recognized as two of the United Nations Sustainable Development Goals: #2: “achieve food security,” and #5: “achieving gender equality,” and includes Target 5.2, “eliminate all forms of violence against women and girls” [3]. Food insecurity occurs when a person lacks regular access to sufficient, nutritious food, thereby impeding their growth and development [4]. Several factors increase the risk of food insecurity worldwide, including climate change, poverty, financial distress, war, and ecosystem disturbances [5]. These risks are further compounded by intersections of multiple social and structural factors, which increase an individual’s vulnerability [6, 7]. The prevalence of food insecurity was exacerbated by the COVID-19 pandemic [8]. In 2021, more than 939 million women worldwide suffered moderate to severe food insecurity [4]. Compared with men, women are more likely to experience food insecurity within the same household according to a study done in Nepal [9]. In the United States (U.S.), food insecurity worsened during the COVID-19 pandemic due to unemployment [10] and economic restrictions, as well as concerns about food and resource shortages. Lockdowns during the pandemic led to delays in meat and food processing, which contributed to a surge in food prices due to the shutdown of agricultural food processing worldwide [5].

Food insecurity can impact women’s health and well-being. Globally, nine million people die annually because of hunger and illnesses associated with hunger [11]. Physical and maternal health impacts of food insecurity include noncommunicable diseases [12], gestational diabetes, preeclampsia, and preterm birth [13]. Food insecurity has also been found to also worsen mental health, such as generalized anxiety disorder, stress, and post-traumatic stress symptoms [14]. Food insecurity can trigger the risk of suffering from GBV, such as IPV, family violence, and child marriage [15, 16]. Violence related to food can also occur when a household lacks food. This lack of food is associated with increased household stressors and conflicts, as well as a decreased ability to cope with stressors. These stressors and conflicts are associated with increasing control behaviors by perpetrators of violence [17].

GBV is considered a public health issue with a high prevalence worldwide as well as an alarming increase in incidence during the pandemic [18, 19]. For example, GBV affected an estimated one in three women worldwide, approximately 736 million women [20]. Several countries reported a substantial increase in GBV, with some regions reporting higher helpline call volumes and increased service use during the COVID-19 pandemic. There was a 48% increase in GBV helpline calls in Spain [21], a 50–60% increase in two countries from the Eastern Mediterranean Region [22], and a 775% increase in the African continent, such as Kenya [23]. Lockdowns related to COVID-19 increased risk factors for GBV at the individual and social levels because of economic insecurity, increased alcohol use, school closures, and decreased access to health and social services for victims [19, 24]. One in two women reported experiencing GBV during the pandemic [25] Lockdown restrictions and social distancing increased isolation, hindered abuse reporting, and exacerbated challenges such as inadequate transportation, food insecurity, and digital divides [24].

Given the potentially dire health consequences of food insecurity and GBV, there is a need to understand how the pandemic impacted the association between GBV and food insecurity in women. Although the literature has established that food insecurity and GBV were associated during the COVID-19 pandemic, a gap in understanding these two public health issues remains. Existing studies and reviews often assess food insecurity and GBV separately, and few have examined the pathways linking them during the COVID-19 pandemic across the socio-ecological level and through an intersectional lens. To address this gap, this systematic review maps and summarizes the existing studies on the association between food insecurity and GBV during the COVID-19 pandemic and explores the socio-ecological factors that influence this association. We focused on women for this work because worldwide evidence shows that women experience disproportionately high rates of both food insecurity and GBV compared with men, and limiting the scope to women allows for a more meaningful synthesis of the available evidence.

Public health practitioners and healthcare providers play essential roles in addressing food insecurity and GBV through screening, providing care, and advocating for people who suffer from food insecurity and GBV [26]. Public health practitioners, such as public health nurses, can also play a key role at the policy level in developing and implementing long-term solutions for addressing food insecurity and GBV [26]. Violence against women (VAW) service providers and organizations, social workers, and GBV advocacy groups also have a critical role in preventing and addressing food insecurity and GBV. These organizations offer crisis intervention, safety planning, access to shelter, and case management [27]. Strengthening collaboration between these sectors can better serve women’s needs, improve the referral pathway, enhance the quality of care, and ensure that actions and interventions address food insecurity and GBV.

### Theoretical framework

The intersectionality framework and the social-ecological model (SEM) guided this study in understanding the association between food insecurity and GBV. The intersectionality framework offers a profound understanding of how multiple social identities, such as race, gender, socio-economic status, ability, and immigration status, interact with and influence an individual's experiences of discrimination, oppression, and vulnerability [6, 28]. The intersectionality framework is considered an inclusive lens for addressing social inequality and combating vulnerability to injustice [6, 28]. SEM aims to understand how humans develop and interact with surrounding circumstances [29]. For this systematic review, we adapted the SEM of the violence prevention model from the Centers for Disease Control and Prevention website [2]. The SEM includes four complex spheres: individual, relationship, community, and societal. The individual level focuses on human backgrounds and biological factors that affect humans, such as education level, age, attitudes, and beliefs. The relationship level includes how an individual interacts with close relationships, such as with partners or family members. These relationship dynamics can influence a person's vulnerability to perpetrating or experiencing violence and may contribute to food insecurity. The community level involves the social setting surrounding the individual, such as the workplace, school, or neighbors; social settings could influence people's risk of living or surviving violence and increase vulnerability to food insecurity. The societal level covers the broader context of how the environment in society could support or prevent violence and food insecurity, for example, social norms that justify violence and create gender inequalities in the community and laws and policies [2]. Combining the intersectionality and SEM framework enables a multi-level, equity-informed comprehension of how food insecurity and GBV intersect. While the SEM highlights the impact of the interpersonal and social spheres on these issues, the intersectionality framework provides insight into the overlapping of social positions and systems of vulnerability that frame women’s lived experiences of food insecurity and GBV, ultimately enabling researchers and healthcare providers to create more holistic and sensitive interventions and programs.

### Aim

The purpose of this systematic review was to explore the association between GBV and food insecurity among women during the COVID-19 pandemic, using both intersectionality theory and SEM to examine how multi-level factors and overlapping identities influence this association. Also, we aim to inform public health practitioners, VAW organizations, social workers, and GBV advocacy groups in their development of strategies and actions to reduce health disparities, promote equity, and develop more responsive support services. For example, VAW organizations, community-based, and policy-level interventions should be designed to promote women’s health and well-being globally. Integrating strategies across the individual, relationship, community, and societal levels hold promise for strengthening the effectiveness of food insecurity and GBV interventions.

## Methods

### Design

A systematic review design was employed, utilizing the Preferred Reporting Items for Systematic Reviews and Meta-Analyses (PRISMA) were used to inform the selection strategy of the studies [30]. The Quality Assessment Tool for Studies with Diverse Designs (QATSDD) was used to evaluate the included studies [31]. The systematic review protocol in this review has not been registered in any public registry; therefore, to enhance methodological transparency, Supplementary Tables 1 and 2 present the PRISMA checklists for the full systematic review and for the abstract [30].

### Search method

Five databases were searched in November 2025, including APA PsycINFO, CINAHL, MEDLINE, PubMed, and Web of Science. A health sciences research librarian helped identify the search terms and refine and validate the search strategy. The search terms included (Food insecurity OR food access OR hunger OR malnutrition OR food scarcity OR nutritional deprivation OR food security OR food deprivation OR food availability) AND (gender-based violence OR GBV OR family violence OR family abuse OR intimate partner violence OR IPV OR intimate partner abuse OR dating violence OR spouse abuse OR spousal abuse OR spousal violence OR domestic violence OR domestic abuse OR partner violence OR marital violence OR violence against women) AND (Coronavirus OR coronavirus OR corona virus OR covid19 OR covid 19 OR nCoV OR CoV 2 OR CoV-2 OR sarscov2 OR 2019nCoV OR lockdown) AND (women OR female). Manual searching of the reference lists of the included studies was not performed. Supplementary Table 3 presents all the search terms used for each database.

### Study eligibility

The inclusion criteria for this review were as follows: 1) population – studies focused on women; 2) exposure to GBV including IPV during the COVID-19 pandemic; 3) comparison of food insecurity and its association with GBV; 4) peer review, original research data-based quantitative and qualitative studies, and mixed-methods research; 5) studies should be in the English language; and 6) data collection conducted during the COVID-19 public health emergency period. According to the World Health Organization, COVID-19 was declared a Public Health Emergency of International Concern on January 30, 2020, and this declaration was lifted on May 4, 2023 [32]. Therefore, the included studies were those with data collection conducted between January 2020 and May 2023. Studies were excluded if they investigated only one of the targeted variables (food insecurity or GBV) or if they were discussion papers, reviews, or editorial papers. Two independent reviewers screened each study for eligibility for inclusion in the review. Any conflicts or disagreements were resolved by consulting the senior author.

### Quality appraisal

A quality appraisal was conducted using QATSDD to evaluate the strengths and limitations of the included studies. The QATSDD is suitable for assessing the quality of quantitative, qualitative, and mixed-method studies. It consists of 16 items, with scores ranging from 0 to 3 for each item [31]. Two authors independently performed quality appraisals. After the first five studies were assessed, the reviewers met to discuss discrepancies and resolve differences. Following all appraisals, the authors met to reach a consensus on the overall quality assessment. The following calculation was used to compare the quality appraisal scores: the total possible score for a mixed-method study was 48, and 42 for quantitative and qualitative studies. Thus, the total score divided by the maximum possible score for each study was obtained and converted to a percentage (e.g., 38/42 = 90.5%).

### Data extraction and data synthesis

The studies were imported into EndNote, and two independent reviewers (HA and EA) independently conducted full text review for data extraction and data synthesis of the 32 studies. Data extracted were entered into an Excel sheet, which included the following: authors, publication year, country, study purpose, study design, target population, sample size, and study findings. We utilized a blended thematic analysis approach that integrated both deductive and inductive coding, grounded in intersectionality theory and the SEM. Reviewers systematically extracted data examining the bidirectional relationship between food insecurity and GBV during the COVID-19 pandemic, specifically how food insecurity increased women’s risk and experiences of GBV and how GBV, in turn, contributed to worsening food insecurity. Consistent with the SEM, deductive a priori coding categories were applied across the individual, relationship, community, and societal levels. At the same time, the inductive development of additional themes and subthemes was permitted to avoid constraining emergent findings within the predefined coding categories.

The evidence was then analyzed via summary tables, descriptive statistics, and thematic analysis. We conducted thematic analysis guided by the method described by Hennik and colleagues [33]. Two independent researchers (HA and EA) read the findings multiple times to familiarize themselves with the data before developing the initial codes. We developed an initial coding framework informed by the intersectionality framework and SEM to guide data synthesis. We operationalized intersectionality by coding social identities (e.g., immigration status) alongside structural or contextual factors (e.g., mental health challenges, employment issues) as separate categories and then examining how these dimensions intersected to shape women’s experiences. We used an intersectional analytic lens to understand how multiple identities and structural inequities converged within and across SEM levels. The codes were then organized into broader conceptual categories and iteratively refined into themes. Each theme was clearly defined and interpreted in relation to the review's aim and the guiding theoretical frameworks, allowing us to capture overlapping vulnerabilities. Coding discrepancies were resolved through iterative discussions and, when needed, review by a senior author. After coding was finalized, the team collaboratively refined and organized themes within and across SEM levels. As this review did not conduct a meta-analysis, the data were synthesized thematically, and no imputation was performed. This approach was employed due to the heterogeneity of study designs, outcomes, and measures across the included studies. Potential sources of heterogeneity were identified, including study designs, outcomes, populations, and countries.

## Results

The associations between food insecurity and GBV during the COVID-19 pandemic could be explained through several interrelated factors at the individual, relationship, community, and societal levels. The individual level encompassed mental health conditions and immigration status. The relationship level involved household instability. The community level included poverty and limited employment opportunities. The societal level encompassed restrictive policies during the COVID-19 pandemic and cultural norms. The detailed characteristics and findings of each study are presented in Table 1.

**Table 1:** Study characteristics and findings

Authors/Year/Country	Research Aim	Research Design	Population/Sample size	Main Outcomes	Quality Appraisal
Abrahams et al. (2022) [34] South Africa	To assess the association between common mental disorders (CMDs), food insecurity, and domestic violence	Cross-sectional survey	Pregnant women (*n* = 885)	• The prevalence of common mental health conditions (CMDs) [depression and anxiety] increased with severe food insecurity, physical, psychological, and sexual abuse, increased crime in the community, and diminished food availability in the household during the lockdown • Experiences of psychological abuse were associated with severe food insecurity, diminished food availability in the household during lockdown, less household income, and increased crime in the community	76.2%
Abrahams & Lund (2022) [35] South Africa	To assess the association of risk factors with the prevalence of food insecurity and CMD	Cohort study	Pregnant women (at baseline* n* = 859, follow up *n* = 635)	• Food insecurity was associated with racial identity, psychological distress, domestic violence, poverty, and unemployment • Women who have mental conditions reported high levels of psychological and/or physical abuse, and food insecurity	78.6%
Bhandari et al. (2023) [36] India	To assess the experiences of IPV forms and how these forms are related to nutritional outcomes	Secondary analysis	Women (*n* = 60,622)	• Experiencing high levels of physical IPV was associated with women’s nutritional outcomes • The experiences of high levels of physical and sexual violence impact on women’s nutritional outcomes, such as being undernourished and underweight	64.3%
Bonsu et al. 2025 [37] Canada	To investigate food insecurity experiences among women who experienced violence accessing supportive services during the COVID-19.	Qualitative study	Women (*n* = 10)	• Power imbalance in the relationship, including physical restrictions, prevents women from accessing food from their husbands and mothers- in-law. • Even though the money was under the control of the partner, women were responsible for providing food for themselves and their children. • Women who were vulnerable with precarious immigration status experienced exploitation and control by their partners, including their access to basic needs such as food and to feed themselves and their children which contributed to worse experiences of violence and disempowered women. • During the pandemic, abused women who were in shelters mentioned that the food was poor quality and the amount of food was insufficient. • The rules related to food during COVID-19 were harmful to their ability to heal from their abuse. • The lines were long in the shelter to get food for social distancing. • The transition out of shelters placed women in financial insecurity, including a lack of housing and employment, and the unavailability of childcare supports, which prevented women from accessing food and their sense of independence and autonomy. • Women who had unstable immigration status and left their partners were not qualified for government financial support. • Some shelters during the pandemic had reduced programming or the ability to provide food support.	76.2%
Brody et al. (2023) [38] Cambodia	To assess the association between income loss during COVID-19 and GBV among female entertainment workers	A cross-sectional study	Women (*n* = 207)	• Women who lost total income were more likely to experience GBV and food insecurity.	66.7%
Brandhorst & Clark [39] (2022) U.S.	To assess the prevalence of food insecurity and the relationship between food insecurity and women’s well-being	Quantitative paper	Abused women (*n* = 26)	• Food insecurity was associated with experiencing abuse • Food insecurity was associated with depression, PTSD, disability, concentration, lack of hope, and decreased well-being	69%
Chua et al. (2024) [40] Cambodia	To explore the factors that impact the mental well-being of female entertainment workers during COVID-19.	Quantitative study	Stakeholders (*n* = 27)	• Income cuts led to an increase in stress and tension, and a turn to GBV. • The mental health stressors hindered women's ability to work and earn an income.	52.8
Cochran et al. (2022) [41] U.S./Michigan	To assess whether economic hardship predicts IPV experiences	Longitudinal study	Pregnant women (*n* = 294)	• Economic hardship (food insecurity & money problems) predicted IPV • Economic hardship was positively associated with IPV	76.2%
Correia et al. (2024) [42] Brazil	To assess the determinants of domestic violence and to explore the patterns of domestic violence over time	Longitudinal cohort study	Postpartum women (*n* = 331) in 3rd round and (*n* = 322) in 4th round	• Domestic violence was associated with CMD, low education level, and food insecurity • Women who headed the household had twice the susceptibility of experiencing domestic violence	71.4%
Diaz et al. (2022) [43] U.S./New York	To investigate the effect of lockdown on adolescent and young adult females	Cohort study	Adolescent and young adult women (*n* = 417)	• Participants reported engaging in sexual activity; exchanging pornography for cash, drugs, food, or shelter • Several living conditions before and during COVID-19 have contributed to increasing chances of GBV, such as lack of access to food, water, healthcare services, and medication	60%
Duby et al. (2022) [44] South Africa	To assess the socio-economic and mental health effects of COVID-19 on adolescent and young adult women	Mixed method	Adolescent and young adult women (surveys *n* = 515, interviews *n* = 50)	• 20% of participants reported that their relationships with family members had worsened • 14.6% of participants cited increased violence in their homes and shared that they were worried about abuse • Family relationships worsened during the pandemic • Experiencing hunger, unemployment, intense dissatisfaction, and fears about money increased, which increased conflict and fighting • Food insecurity was associated with mental health outcomes • Family segregation during lockdowns caused a breakdown in family dynamics and catalyzed intrafamilial violence	83.3%
Getinet et al. (2022) [45] Ethiopia	To assess the prevalence of IPV and its related factors	Multistage community-based cross-sectional study	Women in the reproductive age group (*n* = 845)	• IPV was associated with being married, having no formal education, having > 3 children, food insecurity (2 times), having life-threatening events, moderate social support, and depression	85.7%
Giacomini et al. (2023) [46] Brazil	To assess the prevalence of IPV and its related factors and to explore the association between IPV and CMD	Cross-sectional survey	Women (*n* = 479)	• The prevalence of CMD among women who experienced IPV was 41.3%, compared to those who did not, 20.7% • IPV was associated with CMD • Women who had experienced IPV had twice the odds of experiencing CMD • IPV was common among those who experienced food insecurity, loss of jobs during COVID-19 (twice), and in women whose fathers of their children did not exist at home or did not visit the child • Food insecurity, diminished food availability, and job loss during COVID-19 were predictors of CMD	66.7%
Hamadani et al. (2020) [47] Bangladesh	To investigate the impact of the COVID-19 lockdown on women and their families	Time-series design	Women and their families (*n* = 2424)	• Women living with their husbands reported an increase in IPV during lockdowns • Food insecurity was high among families with a parent with an unskilled job	69%
Hartmann et al. (2023) [48] South Africa	To assess the impact of COVID-19 on GBV (community, household, IPV) and examine the association between GBV and mental health	Cross sectional-study	Young people with or without HIV (women *n* = 373, men *n* = 159)	• Community violence increased with living in informal housing, being food insecure, and having an intimate partner during lockdown • Being a man, having CMD, COVID-related stressors, and being food insecure were associated with an increased risk of experiencing household violence • Living in informal housing, food insecurity, and household violence are risk factors for GBV	81%
Heck et al. (2025) [49] U.S./New York	To assess the predictors of psychological distress among college students	Longitudinal study	(T1 = 556, T2 = 334, T3 = 221, and T4 = 169)	• 96% were cisgender women. • Feeling distressed was high among those who had high food insecurity and experienced violence.	52.8%
Humphries et al. (2022) [50] South Africa	To explore the impact of COVID-19 on the socio-economic and health care access among women living with HIV or at high risk of acquiring HIV.	Cross-sectional survey	(*n* = 2812)	• Women who experienced GBV were more likely to experience food insecurity.	76.2%
Huq et al. (2021) [51] India	To recognize how the pandemic affected women’s needs and experiences	Qualitative interviews	(*n* = 586)	• The combination effect of insecurity and violence was a stressor. • The COVID-19 policies affected power imbalances and inability to find relief outside the home. • Partners controlled the money from women and restricted their movements in the house, isolating women socially and denying them food. • The primary source of stress was the increased burden that fell on women, including housework and children being off school, leading to fatigue and frustration from gender norms.	57.1%
Jacob et al. (2024) [52] Tanzania	To assess the role of healthy lifestyle factors (diet, sleep, and exercise) in IPV perpetration	Cross-sectional survey	Young men (*n* = 1002)	• Men with poor food variety had 57% higher odds of committing sexual IPV • Stress increased with living in small spaces of informal settlements	76.2%
Karp et al. (2021) [53] Kenya	To determine the influence of COVID-19 restrictions on girls and young women’s relationship experiences	Mixed method	Adolescent girls and young women (*n* = 756)	• Adolescent girls reported they became dependent on their partners’ incomes instead of their family’s income • 20% of participants reported that their relationships with family members had worsened • Relationships were strained by COVID-19 mitigation measures • School closures accelerated marriage timelines, and economic hardships increased the risk of early pregnancy	77.1%
Khofi et al. (2025) [54] South Africa	To understand the relationship between food insecurity, gender, migration, and IPV, with restricted access to sexual and reproductive health services	Qualitative interview	Women (*n* = 15)	• Women's partners restricted them from economic resources and their movements. • Immigrant women without legal documentation started working in sex work because of the financial pressure, limited employment choices, and to meet basic needs, including food.	64.3%
Lindau et al. (2021) [55] U.S.	To investigate the association between changes in health-related socio-economic risks (HRSRs) and mental health	Cross-sectional survey	Women (*n* = 3200)	• Among the list of health-related socioe-conomic risk factors, pre-pandemic food insecurity was most likely to be exacerbated, which led to those not food insecure pre-pandemic becoming newly food insecure in the early stages of the pandemic • Women tend to suffer higher rates of anxiety, depression, and traumatic stress in times of crisis because of worrying more about household needs than their male counterparts	61.9%
Mahlangu et al. (2022) [56] South Africa	to examine the impact of IPV and child abuse experiences during lockdown and its effects	Qualitative	Women and men adults (*n* = 37)	Food insecurity caused by socio-economic conditions during COVID-19 and the lockdown led to: • Worsened experiences of emotional and physical violence • Worsened verbal aggression toward both women and children During the COVID-19 pandemic and lockdown: • Low socio-economic-status households experienced increased stress, arguments, and conflict due to reduced spending and fewer resources • Women who took responsibility for finding resources to get food and care for the household • Men felt pressure to provide for their families but preferred not to talk about their (lack of) salaries and became aggressive • Lack of psychosocial support, closure of psychosocial support services, and isolation from social networks during lockdowns left people with no coping mechanisms • Participants reported difficulty managing conflict and arguments in the home • Some participants described using self-calming techniques to cope with the stress of lockdowns and apologizing to spouses to manage conflict • Women in low socio-economic status groups were at higher risk for experiencing emotional abuse from their male spouses and intimate partners because of a lack of food and other basic household necessities • Some men used violence as a stress reliever for their internal feelings • A sense of lack of control during confinement made men feel their masculinity would be doubted by their partners • Food insecurity resulted from unemployment and economic loss	77.4%
Murray et al. (2023) [57] Brazil	To assess the experiences of mental health problems during the COVID-19	Prospective, population-based birth cohort study	Children and caregivers (*n* = 2083)	• Mental health conditions increased during the pandemic compared to prior to the pandemic • Poorer families were more likely to suffer financial pressure, food shortages, increased conflict in relationships, parenting problems, and child worries about food availability during COVID-19 • These difficulties lead to increased mental health problems • Child worries about food availability during COVID-19 were associated with emotional problems	77.4%
Nuwematsiko et al. (2022) [58] Uganda	To investigate socio-economic and health consequences because of the COVID-19 pandemic and the mitigation measures among slum dwellers in Kampala.	Mixed methods	Female (*n* = 282) and male (*n* = 143)	• Domestic violence increased during the pandemic due to financial distress and loss of jobs. • Some families were broken up due to domestic violence, because of the increasing need for basic needs such as food. • Women were beaten by their husbands because they did not afford food to their families.	79.2%
Pinchoff et al. (2021a) [59] Kenya	To examine the economic, social, and health-related impacts on women	Longitudinal cohort study	Urban adults (*n* = 2009)	Households experienced: • worsened food insecurity • Worsened violence • have forgone health services • Women were more impacted than men • Participants reported skipping a meal the previous week • Men were more likely to skip meals when their households were larger and had more people living there • Women reported higher levels of hostility, fighting, violence, or worry that a partner would hurt them	76.2%
Puri et al. (2023) [60] Nepal	To explore the factors associated with IPV and how IPV was impacted by food insecurity and COVID-19	A longitudinal study	Newly married Women (*n* = 200)	• COVID-19 and food insecurity were associated with an increase in IPV • Young, newly married women, increased length of the marriage, women who had low education level, and pregnancy were more likely to have a high risk of experiencing IPV • Protective factors of experiencing food insecurity were women had paid jobs	69%
Ringwald et al. (2023) [61] Kenya	To understand the power dynamics in the influence vulnerability in IPV and HIV	Qualitative study	Women (*n* = 56) and 32 men (*n* = 32) and key informants (*n* = 10)	• Experiencing poverty, such as food insecurity and lack of money to pay expenses, is a key driver to experiencing IPV • Some women and men fled from poverty through love relationships, transactional sex, and sex work to cover expenses, leading to amplified IPV • No support was available to navigate gender roles • Financial pressure led to increased stress and psychological pressure on spouses and intimate relationship problems, which increased the odds of experiencing IPV • Gender expectations were disturbed • Alcohol and drugs were used during the pandemic to cope with stressors • Alcohol and drugs were cheaply and available, leading to exacerbate IPV	85.7%
Shimizu et al. (2023) [62] U.S.	To understand the role of food behaviors in intimate relationships	Qualitative study	Adults (*n* = 61)	• Several personal, community, and organizational factors contributed to elevated violence during the pandemic, such as household food insecurity • The role of food behaviors served in promoting unequal relationships • Male partners did not appreciate or ignore women's cooking because they felt frustrated with their partners' food preparation • Food and food behaviors are likely key drivers of tension, stress, or violence • Lack of food can trigger anger and violence	85.7%
Singh et al. (2021) [63] Nepal	To assess food insecurity during the pandemic among people from the disadvantaged community and low-income families	Qualitative	Urban and rural adults (*n* = 41)	• Loss of income resulted in frustration, anxiety, and depression, especially among men who could not provide food for their families • The increased risk of mental health conditions and GBV is more common among low-income and vulnerable families • Economically disadvantaged people experienced increased food insecurity • Only those with political connections and affiliations benefited from food relief packages • People developed various strategies for coping with the situation	85.7%
Ujah et al. (2024) [64] Nigeria	To assess the association between food insecurity and IPV	Population-level cross-sectional analysis	Women (*n* = 23,200) and men (*n* = 7087)	• 76.6% had limited food variety, and 66.9% of participants skipped a meal because of economic hardship • 59.3% of participants were out of food, and 50.4% were hungry because of a lack of money to get food • Severe household food insecurity was associated with the acceptance of physical IPV against women by 11-fold	83.3%
Vahedi et al. (2023) [65] Brazil	To assess the impact of structural violence and GBV during COVID-19	Qualitative study	Service providers (*n* = 12)	• During COVID-19, unmet basic needs led to increased conflict, tension, and violence in the household • Loss of income leads to increased sexual exploitation among children • Structural violence was amplified during COVID-19 and turned into increased VAW and children • School closures forced children to spend more time with family members, increased the risk of violence, and hindered children from getting supplies and social support from the school • School closures limit the ability of mandatory reporting of child abuse and neglect and appropriate referrals • Child marriage and early marriage were increased during COVID-19	85.7%

### Description of the included studies

**Fig. 1:**
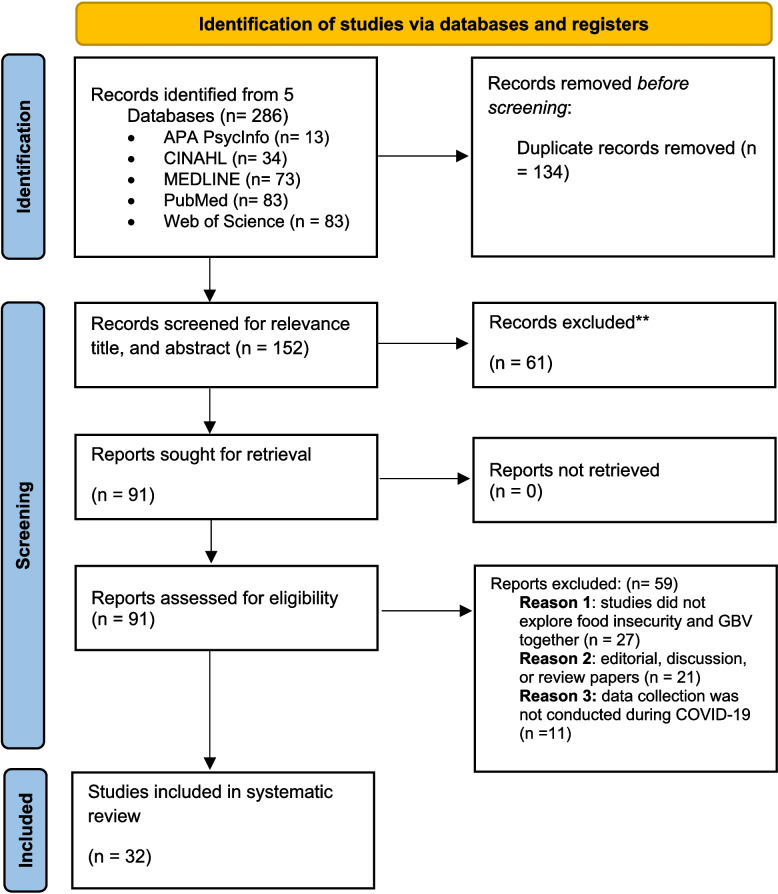
PRISMA flow chart. From: Page et al. (2021) [30]. For more information, visit: https://doi.org/10.1136/bmj.n71

A total of 286 articles were retrieved in the initial search, and 134 duplicate studies were removed. A total of 152 articles were moved to initial screening after the titles and abstracts were reviewed. Ninety-one studies were subjected to a second screening by two independent reviewers, who read the full text, and a final sample of 32 articles was included in this systematic review. Studies were excluded during the second screening because they did not assess the association between food insecurity and GBV together, because the studies were not primary research, or because the studies did not collect data during the COVID-19 pandemic. Figure 1 delineates the PRISMA guidelines for the study selection process.

The included articles were published between 2020 and 2025. Two-thirds of the studies included in this review were quantitative-based. Among the twenty-one quantitative studies included, eleven were cross-sectional studies, five were longitudinal studies, three were cohort studies, one employed an interrupted time series design, and one was a secondary analysis study. Additionally, nine qualitative articles met the review criteria and were included. Finally, three articles were mixed-methods studies. We included studies that focused on women, regardless of their background or identity. For example, we included studies involving adult women, pregnant and postpartum women, women of reproductive age, abused women, young women with or without HIV, female entertainment workers, and sex workers. Some of the studies included in this systematic review also included young men who perpetrated GBV against women, as well as service providers who discussed perspectives related to GBV and food insecurity against women. The studies were primarily conducted in low- and middle-income countries, including South Africa (*n* = 7), Kenya (*n* = 3), Brazil (*n* = 4), India (*n* = 2), Nepal (*n* = 2), Ethiopia (*n* = 1), Bangladesh (*n* = 1), Nigeria (*n* = 1), Tanzania (*n* = 1), Cambodia (*n* = 2), and Uganda (*n* = 1). However, a few included studies were conducted in the U.S. (*n* = 6), and one included study was conducted in Canada. The sample sizes ranged from 10 [37] to 60,622 participants [36] across all studies. In quantitative studies, the association between food insecurity and GBV was reported using measures such as mean differences, correlations, odds ratios, relative risks, and 95% confidence intervals. In qualitative studies, this association was identified through themes describing how food insecurity and GBV were linked.

### Quality appraisal

The mean score of the quality appraisal for all studies was 73.4%, ranging between 52.8% and 85.7%. The mean score of the quality appraisal among the quantitative studies was 69.5%, ranging from 52.8% to 85.7% (*n* = 21), while among the qualitative studies, it was an average of 77.2%, ranging from 57.1% to 85.7% (*n* = 8). Across the mixed-methods studies, an average score of 79.9% was found, ranging from 77.1% to 83.3% (*n* = 3). More specifically, the strengths of the studies' quality were as follows: 91% (*n* = 29) had a clear description of the study aims/objectives, 100% (*n* = 32) had a clear description of the research setting, 100% (*n* = 32) had a representative sample of the target group with a reasonable size, 88% (*n* = 28) had a clear description of the data collection procedures and rationale for the choice of data collection tools, 63% (*n* = 20) had a clear and detailed report of the recruitment data, 94% (*n* = 30) had a good fit between the research question and method of data collection, and data analysis, and 91% (*n* = 29) critically discussed the studies' strengths and limitations.

The majority of the limitations of the studies' quality were as follows: of the 32 total studies, 78% (*n* = 25) lacked a defined conceptual/theoretical framework, 68% (*n* = 21) lacked clear evidence of sample size consideration in terms of analysis, 84% (*n* = 27) lacked clear evidence of testing/reporting reliability and validity of relevant measurement tools, and 94% (*n* = 30) lacked evidence of using a pilot study or potential participant engagement to inform the design of the study.

### Associations between food insecurity and GBV

There was a noticeable elevation in the incidence of food insecurity and GBV during the COVID-19 pandemic. The forms of GBV identified in the included studies were domestic violence and IPV, expressed as behaviors such as physical, psychological/emotional, controlling, sexual, and economic violence. The majority of the studies in this review revealed an association between food insecurity and GBV. That is, food insecurity and GBV were found to be correlated with one another. For example, women who experienced GBV were more likely to experience food insecurity [50]. Psychological abuse among pregnant women was associated with severe food insecurity [34]. High levels of physical and sexual IPV were associated with nutritional outcomes, such as being undernourished and underweight, with 18.1% of women being underweight [36]. Exposure to IPV doubled the risk of experiencing any level of food insecurity among women of reproductive age [45]. Severe household food insecurity among women was associated with an 11-fold increase in the likelihood of experiencing IPV [64]. The factor that increased the risk for women to experience sexual IPV by men was poor food variety [52]. Married couples who had skipped a meal in the last seven days due to COVID-19 were also more likely to report an increased risk of household violence [59].

**Fig. 2:**
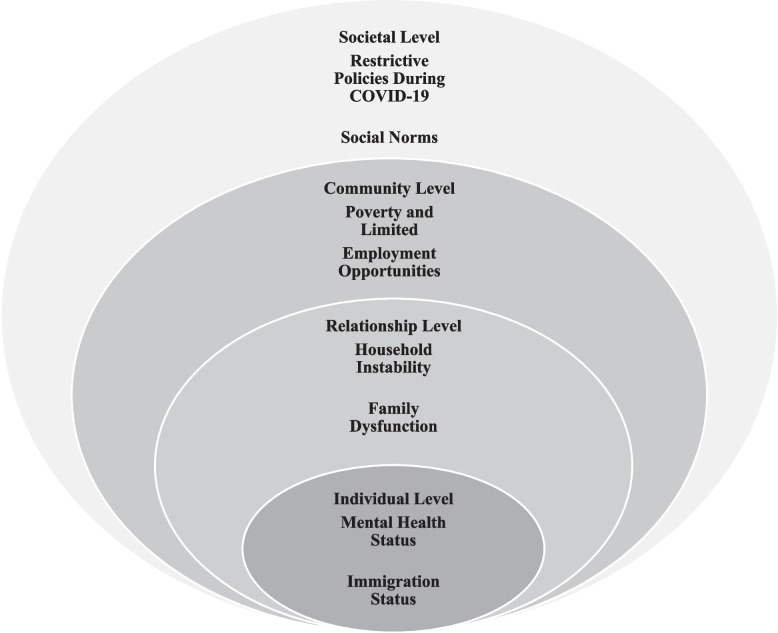
The association between food insecurity and GBV during the pandemic

Applying the review findings to the intersectionality and SEM frameworks, showed that the association between food insecurity and GBV during the pandemic was shaped by several complex, interrelated factors underscored by social structures operating at four levels: the individual, relationship, community, and societal levels. Figure 2 represents the interrelated factors associated with food insecurity and GBV.

#### Mental health conditions

Common mental health disorders (CMDs), such as depression, post-traumatic stress disorder, and anxiety, worsened during the pandemic, along with food insecurity, reduced food availability, and GBV, predicting these CMDs [34, 39, 44, 46]. The mental health impacts were further compounded by the intersections of women's social positions, including socio-economic and immigration status, which interacted with structural inequalities that may intensify their vulnerability to food insecurity and GBV during the pandemic.

Women who felt distressed were also likely to report high levels of food insecurity and experience violence [49]. The combination of insecurity and violence increased stress [51]. Women are more vulnerable to CMDs during crises, as they tend to worry more about household needs than men [55]. Awareness of the link between poor nutrition, mental health, GBV, and general health was limited among women. Other factors contributing to increased women’s vulnerability to GBV during the pandemic were that men experienced frustration, anxiety, and depression due to job loss [63], which increased men’s use of VAW. Similarly, increasing parental problems between children and caregivers was associated with increased mental health problems among caregivers and children [57].

A study among perinatal women showed that women with unique socio-demographic vulnerabilities, such as having CMDs, were at high risk for experiencing food insecurity and GBV [35]. Another study conducted among perinatal women showed that women who had CMDs such as depression and anxiety had a significantly increased likelihood of being severely food insecure, having decreased food availability, experiencing different forms of abuse (e.g., psychological, physical, or sexual abuse), and experiencing increased crime in the community [34]. Women who had experienced IPV had twice the odds of experiencing CMD [46].

Other factors contributing to women's experience of food insecurity and GBV included some men used violence as a stress reliever for their internal feelings [56]. One study found that being a man, having CMD, experiencing COVID-19-related stressors, and being food insecure were associated with an increased risk of women experiencing household violence [48]. COVID-19-related stressors increased the likelihood of a lack of coping skills to manage men's stress and anxiety. For example, some communities in South Africa used substances as a coping mechanism. The lack of psychosocial support and isolation from social networks during lockdowns left people with limited coping mechanisms to manage their stress and anger [56]. Individuals also reported difficulty in managing conflicts and increased arguments at home [49, 55]. In contrast, others described using self-calming techniques to cope with the stress of lockdowns and apologizing to spouses to manage conflict [56].

##### Immigration status

Immigration status, another dimension of one’s social position, was observed to intersect at the individual level with the health risks and vulnerabilities experienced by women [37, 54]. Women who were vulnerable to precarious immigration status experienced exploitation, threats, and control by their partners, including hindering women from accessing basic needs such as food. Also, immigrant women’s partners exploited their situations and at times forced them to find food to feed themselves and their children, which contributed to worse experiences of violence and disempowered women [37]. Women in Canada who had unstable immigration status and left their partners were not qualified for government financial support [37]. An example of how immigration status intersected within a specific country, is that immigrant women in South Africa who were without legal documentation began engaging in sex work due to financial pressures, limited employment choices, to meet their basic needs, including food [54].

#### Relationshipslevel 

##### Household instability

During the pandemic, episodes of food loss in households and behaviors surrounding food availability and food preparation contributed to promoting unequal relationships, role confusion, and household instability by triggering anger and violence [62]. For example, some women expressed that their partners lacked responsibility because they did not provide food. In other cases, women were beaten by their husbands because it was difficult to provide food to their families [58]. For some women’s partners, frustration with food preparation as well as a lack of appreciation for or lack of recognition of women's cooking efforts occurred [62]. Some men shifted food-related responsibilities completely to women, believing that women alone planned for family meals when money was scarce, which contributed to confusion and household instability [56].

Women who experienced IPV mentioned that power imbalances in intimate relationships had a role in household instability and food insecurity. Power imbalance often led to imposition of physical movement restrictions, which prevented women from accessing food from their husbands and family members [37]. Additionally, the money needed to purchase food was under the control of the partner; thus, women were responsible for providing food for themselves and their children without accessing the financial resources of their partners [37]. That is, partners not only controlled the money, but also restricted the movements of women within the house, isolated women socially, and denied them food [48, 55].

How a household was structured and maintained also played a role in the association of GBV with food insecurity. That is, women who lived with their husbands reported an increase in IPV during pandemic lockdowns [47]. Women who identified as a primary adult responsible for heading the household during the COVID-19 pandemic had twice the susceptibility to experiencing violence compared with households headed by partners or grandparents [42]. IPV was most common in women whose fathers of their children did not live in the same home or did not visit the child [46]. Younger participants experienced increased community violence^1^ with living in informal housing, being food insecure, and having an intimate partner during lockdown [48]. Sustaining the family was associated with increased stress and psychological pressure on spouses and intimate relationship problems, which increased the likelihood of experiencing IPV [61].

Household stability was also impacted because some men felt frustrated and stressed when staying at home. They felt that their partners did not perform their household duties, which increased the likelihood of experiencing verbal abuse. Some women reported that their partners frequently made comments on trivial things, which exacerbated problems between them [56].

##### Family dysfunction

Along with the impacts on mental health status, immigration-related issues, and gendered power dynamics, the pandemic also had an impact on family structure. The pandemic disrupted household roles and contributed to family dysfunction. Because families are embedded within broader social, cultural, and historical contexts that shape their capacity to respond to crises and maintain stability, it is important to understand these layered influences during the pandemic [44]. Breaking down of the family unit during lockdowns occasionally contributed to a breakdown in family dynamics and catalyzed intrafamilial violence [44]. Family relationships deteriorated during the pandemic because family members were confined to small spaces and experienced hunger, intense dissatisfaction, and financial worries, which exacerbated conflict and fighting [44]. In two studies, 20% of adolescent and young adult women reported that their relationships with family members had worsened [48, 50]. Some families in Uganda were broken up due to domestic violence because of the lack of basic needs such as food [58].

#### Community level

##### Poverty and limited employment opportunities

GBV risk increased in households experiencing economic hardships, such as food insecurity, which was exacerbated by the loss of income during the pandemic. These hardships intersected with individual social positions at the community level, influencing access to essential resources, social mobility, and the ability to adhere to family obligations [38, 43, 46, 56, 61–63]. A lack of food and household necessities can trigger anger and violence [56, 62]. During the pandemic, employment and income declined during the lockdown [41]. People lost their jobs, their salaries decreased, and they were unable to purchase food, which contributed to food insecurity [46, 58]. Experiencing poverty, such as food insecurity and a lack of funds to cover expenses, is a key driver of experiencing GBV [61]. Food insecurity was especially high among families with men who held unskilled jobs [47]. Women who lost total income were more likely to experience GBV and food insecurity than women who had only partially lost their income [38]. Income cuts amplified stress and tension in the household and contributed to GBV against women. The mental health stressors in the household hindered women's ability to work and earn an income [40]. In contrast, protective factors against experiencing food insecurity included having a paid job [60].

Losing income was associated with people feeling frustrated, anxious, and depressed, especially among men, because they could not provide food for their families, which increased the risk of mental health conditions and GBV, such as beating their wives, which was more common among low-income and vulnerable families [63]. IPV was common among women in Brazil who lost their jobs twice during the COVID-19 pandemic [46]. Food insecurity triggered by socio-economic conditions during the COVID-19 and the lockdown intensified and worsened experiences of emotional and physical violence and verbal aggression toward both women and children [56].

Women were vulnerable to food insecurity and GBV because economic distress added extra challenges for men in supporting their families. Men often avoided discussing their (lack of) salaries and became aggressive [56]. Reduced spending, fewer resources, and food shortages during the lockdown increased stress, arguments, and conflict in low-income households [56]. While men were pressured to provide, the responsibility for finding resources to get food and care largely fell on women [56]. Men, seen as providers, faced increased financial pressure, exacerbating stress, anxiety, and violence in the household [56].

Some women and men fled from poverty through love relationships, transactional sex, and sex work for money, food, shelter, and other expenses, which contributed to amplified IPV [61]. During the pandemic, female participants reported more sexual victimization in the form of being forced or coerced into having sex or engaging in sexual activity; exchanging pornography for cash, drugs, food, or shelter; or engaging in sexual activity in exchange for food, shelter, cash, drugs, or other necessities [43, 61].

#### Societal level

##### Restrictive policies during the pandemic

Restrictive COVID-19 policies interacted with intersecting preexisting structural inequities, shaping people’s living conditions, exacerbating vulnerabilities to food insecurity and GBV, and disrupting access to resources during the pandemic. Various aspects of living conditions before and during the COVID-19 pandemic contributed to an increased likelihood of experiencing GBV, including a lack of access to food, water, healthcare services, and medication [43, 65]. The restrictions imposed by the COVID-19 pandemic exposed women to numerous negative consequences. COVID-19 policies exacerbated power imbalances in households and hindered women’s ability to find support outside the home [51]. The government's policies for COVID-19 prevention measures had implications for keeping perpetrators and victims in the same place. Consequently, COVID-19 mitigation measures strained relationships [53]. A lack of resources and the unavailability of network support increased GBV [37]. Access to resources was further restricted by the pandemic's social isolation, which made it difficult for victims to go to a shelter or contact the police [61]. Consequently, victims were forced to stay with their abusers [61]. Compared with men, women were more likely to report an increased risk of violence and to cut out necessary health care [59].

Structural violence, such as a lack of public resources, food availability, and militarization, was amplified during the COVID-19 and turned into increased IPV [65]. During the pandemic, abused women who were in shelters mentioned that the food was of poor quality and that the amount of food was insufficient [37]. Additionally, the lines were long in the shelter when women received food due to social distancing measures. Some shelters during the pandemic reduced programming or the ability to provide food support. The rules related to accessing food during the COVID-19 pandemic were harmful and affected women’s ability to heal from their abuse. When women transitioned out of shelters, they encountered financial insecurity, including a lack of housing, limited employment opportunities and the unavailability of childcare support, which can prevent them from accessing food and undermine their sense of independence and autonomy [37].

Both women and their children were affected by COVID-19 restrictions. School closures during the COVID-19 pandemic forced children to spend more time with family members, increased the risk of violence and sexual exploitation, and deprived children of supplies and social support from schools [65]. School closures limited the ability to make mandatory reports of child abuse and neglect and appropriate referrals. The incidence of child marriage and early marriage also increased during the COVID-19 pandemic [65] and risk of early pregnancy [53]. There was also some reduction in policy implementation due to lockdowns. For example, alcohol and drugs were cheaply available, exacerbating IPV [61].

##### Cultural norms

The role of patriarchal norms intersected with women’s multiple social positions to reinforce existing inequalities and increase the risk of suffering food insecurity during the pandemic. As structural inequalities in communities were exacerbated during the COVID-19, women were disproportionately affected, facing heightened vulnerability to both food insecurity and GBV. For example, cultural norms in India, such as that women must obey their partners and care for the house, amplified GBV during the pandemic [51]. Gender role expectations were disturbed, and the increased burden that fell on women, including housework and children being out of school, increased fatigue and frustration due to gender norms [51, 61]. When women did not meet these expectations during the COVID-19 pandemic, men became frustrated and angry. No support existed to navigate traditional gender roles, such as those that required men to provide financial support to the family and women to be responsible for household chores and childcare [61]. These norms were considered acceptable and normalized behaviors within certain communities. Food and food behaviors were likely key drivers of tension, stress, or violence, such as when people broke ingrained customs surrounding food behaviors [62].

## Discussion

This systematic review expands our understanding of the complex relationship between food insecurity and the global GBV among women during the COVID-19 pandemic, which is important to public health practitioners and healthcare providers. We identified 32 peer-reviewed studies conducted during the COVID-19 pandemic and explored the association between food insecurity and GBV. The COVID-19 pandemic contributed to exacerbating food insecurity and GBV globally. Our findings indicate that the relationship between food insecurity and GBV is driven by complex, interrelated factors across the individual, relationship, community, and societal levels, further exacerbated during the COVID-19 pandemic. The relationship between GBV and food insecurity highlights the need for integrated strategies that address both issues simultaneously by creating initiatives that combat GBV through counseling, shelter, and empowerment while also providing food assistance [16, 66]. These factors are further amplified when considering the intersecting social positions of women, which influence their health outcomes. If these interrelated issues are not addressed, cycles of violence and poverty may continue.

Our findings demonstrate that the relationship between food insecurity and GBV is observed at multiple layers of the SEM rather than within a single domain and is further shaped by intersecting systems of social position and oppression within broader societal structures, which is consistent with the intersectionality framework [28, 29]. Prior evidence has demonstrated that structural determinants of health, such as gender inequality, poverty, and cultural norms, shape both food insecurity and the risk of GBV [66]. Our findings highlight how the combination of these determinants during public health emergencies, such as the COVID-19 pandemic, exacerbated women's vulnerability. Food insecurity worsened mental health challenges at the individual level, while IPV fueled maladaptive coping mechanisms such as substance use patterns during the pandemic [67, 68]. We noted the importance of addressing mental conditions that could be highlighted and exacerbated during public health emergencies.

At the relationship level, disrupted gender roles and increased caregiving demand for women, coupled with challenges to men’s conventional role as providers, led to heightened tension within the household and domestic violence, shaping how gender norms influence health behavior and risk, as reflected in previous studies [69, 70]. Helping men understand and manage their feelings, their roles in families, masculine identities, and cultivating strong interpersonal bonds have all been found to support the enhancement of their psychological status [38]. Women disproportionately bear caregiving and household responsibilities during times of instability, reinforcing traditional gender roles and perpetuating unequal power dynamics [43].

Economic instability, characterized by job losses, reduced incomes, and a scarcity of resources at the community level, aligns with existing evidence linking neighborhood poverty to an increased risk of GBV and food insecurity [18, 66]. At the societal level, restrictive public health policies and weakened systems increased vulnerability to GBV [71, 72]. Collectively, these results indicate that although food insecurity and GBV align with expected social determinants of health standards, the COVID-19 pandemic created distinct interactions and pressures that exacerbated these emergencies.

### Limitations

The findings should be interpreted in light of the study's limitations, including our attempt to include all studies conducted during the pandemic that focused on food insecurity and GBV; some studies may have been overlooked particularly studies published in journals not indexed in APA PsycINFO, CINAHL, MEDLINE, PubMed, or Web of Science, studies published in languages other than English, and new studies published after our final search date. However, the search was conducted systematically across five databases, thereby increasing our confidence in the study findings. We noted that no studies were conducted in certain regions, such as the Middle East and Europe, which may have impacted the interpretation of our findings and limited our ability to generalize them to these regions. Most of the study methodologies employed a cross-sectional design, which may limit the ability to establish causality, and self-report bias may also be a limitation. Additionally, studies focused primarily on healthy and pregnant women, with none of the studies focusing on women living with a disability who may have unique healthcare needs. Future studies should, therefore, be more expansive in focusing on the association between food insecurity and GBV in the lives of other populations with unique needs.

### Implications and recommendations

The findings of this review have implications and recommendations for clinical, policy, and public health in addressing structural inequalities and supporting women’s well-being. These implications are highlighted below.

#### Clinical implications and recommendations

Programs that mitigate economic impacts, such as caregiver income-generating activities, food distribution, healthcare vouchers, and other interventions aimed at economic empowerment, can help alleviate the effects of COVID-19 on mental health [73]. In light of current technological advancements, the effectiveness, safety, satisfaction, and usability of internet-based (eHealth) interventions, such as app-based mobile health (mHealth) apps, in treating mental illnesses and promoting mental health are well supported by the literature [74]. mHealth applications show promise, particularly when combined with social elements and adherence-promoting tactics. In addition to devoting time and energy to creating and executing mental health promotion and prevention initiatives, decision-makers should create a digital strategy for individuals' ongoing mental health care [74].

Interventions to enhance and strengthen relationships by attending to issues of gender equity and its implications for gender roles could serve as preventative measures and help minimize conflict to decrease women's experiences of GBV [75, 76]. Additionally, women's empowerment interventions are crucial in addressing household food insecurity, particularly for female-headed households, including skills development, access to financial resources, and income-generating opportunities, which can help mitigate food insecurity and reduce IPV risk [77, 78]. Supporting couples’ counseling that provides couples-focused interventions addressing healthy communication can also improve relationship dynamics and reduce incidents of IPV [79].

Programs that offer financial assistance, such as cash transfers and food aid, show promise in reducing these risks due to the connection between poverty, food insecurity, and GBV. The Support for Victims, Survivors, and Their Families pillar demonstrates a commitment to expanding access to support and services, including emergency financial assistance, to help people impacted by violence make a safe exit and recover. Expanding broader social programs and supports remains essential to reducing the socio-economic inequalities that underpin GBV risk and prevalence for women [80].

Public health practitioners and healthcare providers have an essential role to play in identifying and addressing the vulnerability of women and their communities to food insecurity and GBV by performing proper screening and, after that, implementing workable interventions. Collaborating with anti-hunger organizations that have expertise in food assistance and addressing insecurity is also important, ensuring that communities are served and supported accordingly [81]. Given that healthcare providers, VAW organizations, social workers, and GBV advocacy groups are often the first line of contact for GBV survivors, they should be given the necessary tools and education to provide primary care support effectively and to connect survivors with additional resources such as shelters, hotlines, social services, mental health services, and legal assistance.

#### Policy implications and recommendations

The findings are crucial considerations for policymakers and stakeholders in the development of future policies and in allocating funding for the provision of services to address the unique needs of populations experiencing the greatest vulnerability to both food insecurity and GBV. Policy changes that emphasize gender equity in social protection programs are needed, ensuring that both men and women are supported during crises to reduce stress and associated violence. Targeted interventions are necessary to question and change the patriarchal norms that exacerbate GBV during emergencies. It is crucial to address cultural norms surrounding gendered food responsibilities, such as cultural expectations about who is responsible for purchasing, preparing, and serving food in households, to reduce the pressures and conflicts that lead to violence in collaboration with local leaders and stakeholders, such as community elders and religious leaders [82]. This can be achieved by encouraging cultural shifts toward more egalitarian relationships through education, highlighting the detrimental effects of strict gender norms and power disparities [83]. Additionally, implementing policies resulted in a reduction in food insecurity. For example, increasing the minimum wage has been shown to significantly decrease household food insecurity in Indonesia and the U.S. [84, 85]. A study conducted in the U.S. reported that implementing higher minimum wages and a robust earned income tax credit decreased food security [86]. Other policies were found to be effective in reducing GBV. For example, in Colombia, regions that implemented GBV policies reported a reduction in physical and sexual GBV from 20 to 16% in 2010 and 2015 [87].

#### Public health implications and recommendations

Applying an intersectionality framework alongside SEM provides critical insight into how structural and systemic factors interact across individual, relationship, community, and societal levels to shape the needs of women during crises [6, 29]. This perspective underscores the role of public health practitioners, operating across various public health contexts, in addressing these intersecting vulnerabilities through equitable, community-centered strategies that extend beyond clinical practice [7]. The findings highlight that school closures during the COVID-19 pandemic decreased children's food availability and increased their vulnerability to violence by depriving them of school-based supplies, support, and mandatory reporting of abuse. The strengthening of child protection mechanisms requires the development of community-based networks to monitor and report abuse and exploitation during emergencies when schools are inaccessible. These include focusing on developing alternative child abuse reporting methods, such as confidential hotlines and digital platforms, and ensuring timely intervention during school closures [88]. Additionally, providing financial assistance to families at risk of pushing their children into early marriage or exploitative situations as well as alleviating the economic pressures that lead to such outcomes can be helpful interventions.

As a community-based target intervention, efforts should focus on raising awareness about the long-term consequences of child marriage and abuse, emphasizing the importance of protecting children during crises. Reintegration programs should be created outside family-based care after the pandemic ends for children who missed school or were forced into marriage during the pandemic, providing access to education and psychological support [89]. Policymakers should strengthen and enforce laws against child marriage and child abuse as well as empower girls by increasing access to education and providing economic support to families [90]. At the school level, teachers and school staff should be prepared with tools to identify, report, and refer to child abuse during remote interactions or virtual learning sessions [91]. Public health practitioners and healthcare providers can be a liaison between schools and policymakers to implement these recommendations and enhance the health of all children.

## Conclusion

The complex relationship between food insecurity and GBV affecting women has been exacerbated and complicated further by the COVID-19 pandemic. GBV and food insecurity are influenced by several intricately linked individual, interpersonal, social, and community factors. Public health practitioners and healthcare providers play a vital role in supporting individuals, families, and communities. The results of this review highlight the need for public health practitioners and healthcare providers to adopt equity-focused interventions that address economic vulnerability and women’s safety. Applying the intersectionality framework and SEM helps illuminate how social positions, including gender, poverty, health status, immigration status, power imbalance, and cultural norms, interact to deepen disparities in both food access and experiences of violence. Combining the intersectionality framework with SEM offers a useful lens for understanding and addressing food insecurity and GBV in an integrated manner.

## Supplementary Information

Supplementary Material 1.

Supplementary Material 2.

Supplementary Material 3.

## Data Availability

Not applicable (this manuscript does not report data generation or analysis).

## Abbreviations

GBV: Gender-based violence
IPV: Intimate partner violence
U.S.: United States
SEM: Social-Ecological Model
QATSDD: Quality Assessment Tool for Studies with Diverse Designs
HCPs: Healthcare providers

## Footnotes

1 Violence occurs between people who are not related and usually happens outside the home [92].
