# Supplementary material for: Food insecurity and gender-based violence against women during the COVID-19 pandemic: a systematic review
Source: BMC Public Health. 2026 Jan 27;26:668. doi: 10.1186/s12889-026-26253-3 (PMC12918138; doi:10.1186/s12889-026-26253-3)
Supplement: Supplementary file 1 — Supplementary Material 1. [file 12889_2026_26253_MOESM1_ESM.docx]

**Supplementary Table 1**

*Search Terms*

|  | PubMed | APA PsycINFO | CINAHL | MEDLINE | Web of Science |
| --- | --- | --- | --- | --- | --- |
| Food Insecurity | “Food insecurity”[MeSH] OR food insecurity OR food access OR hunger OR malnutrition OR food scarcity OR nutritional deprivation OR food security OR food deprivation OR food availability | DE "Food insecurity" OR food insecurity OR food access OR hunger OR malnutrition OR food scarcity OR nutritional deprivation OR food security OR food deprivation OR food availability | MH "Food insecurity" OR food insecurity OR food access OR hunger OR malnutrition OR food scarcity OR nutritional deprivation OR food security OR food deprivation OR food availability | MH "Food insecurity" OR food insecurity OR food access OR hunger OR malnutrition OR food scarcity OR nutritional deprivation OR food security OR food deprivation OR food availability | Food insecurity OR food insecurity OR food access OR hunger OR malnutrition OR food scarcity OR nutritional deprivation OR food security OR food deprivation OR food availability |
| GBV | “Gender-based violence”[MeSH] OR GBV OR family violence OR family abuse OR “intimate partner violence"[MeSH] OR IPV OR intimate partner abuse OR dating violence OR spouse abuse OR spousal abuse OR spousal violence OR domestic violence OR domestic abuse OR partner violence OR marital violence OR violence against women | DE "Gender-Based Violence" OR GBV OR family violence OR family abuse OR DE “intimate partner violence" OR IPV OR intimate partner abuse OR dating violence OR spouse abuse OR spousal abuse OR spousal violence OR domestic violence OR domestic abuse OR partner violence OR marital violence OR violence against women | MH "Gender-Based Violence" OR GBV OR family violence OR family abuse OR MH “intimate partner violence" OR IPV OR intimate partner abuse OR dating violence OR spouse abuse OR spousal abuse OR spousal violence OR domestic violence OR domestic abuse OR partner violence OR marital violence OR violence against women | MH "Gender-Based Violence" OR GBV OR family violence OR family abuse OR MH “intimate partner violence" OR IPV OR intimate partner abuse OR dating violence OR spouse abuse OR spousal abuse OR spousal violence OR domestic violence OR domestic abuse OR partner violence OR marital violence OR violence against women | Gender-Based Violence OR GBV OR family violence OR family abuse OR intimate partner violence OR IPV OR intimate partner abuse OR dating violence OR spouse abuse OR spousal abuse OR spousal violence OR domestic violence OR domestic abuse OR partner violence OR marital violence OR violence against women |
| COVID-19 | “Coronavirus”[Mesh] OR coronavirus OR corona virus OR covid19 OR covid 19 OR nCoV OR CoV 2 OR CoV2 OR sarscov2 OR 2019nCoV OR lockdown | DE "Coronavirus" OR coronavirus OR corona virus OR covid19 OR covid 19 OR nCoV OR CoV 2 OR CoV2 OR sarscov2 OR 2019nCoV or lockdown | MH "Coronavirus" OR coronavirus OR corona virus OR covid19 OR covid 19 OR nCoV OR CoV 2 OR CoV2 OR sarscov2 OR 2019nCoV or lockdown | MH "Coronavirus" OR coronavirus OR corona virus OR covid19 OR covid 19 OR nCoV OR CoV 2 OR CoV2 OR sarscov2 OR 2019nCoV or lockdown | Coronavirus OR corona virus OR covid19 OR covid 19 OR nCoV OR CoV 2 OR CoV2 OR sarscov2 OR 2019nCoV or lockdown |
| Women | “Women”[Mesh] OR women OR female | DE “Women” OR women OR female | MH “Women” OR women, OR female | MH “Women” OR women OR female | Women OR female |
