# Supplementary material for: Food insecurity and gender-based violence against women during the COVID-19 pandemic: a systematic review
Source: BMC Public Health. 2026 Jan 27;26:668. doi: 10.1186/s12889-026-26253-3 (PMC12918138; doi:10.1186/s12889-026-26253-3)
Supplement: Supplementary file 2 — Supplementary Material 2. [file 12889_2026_26253_MOESM2_ESM.docx]

| **Section and Topic** | **Item #** | **Checklist item** | **Location where item is reported** |
| --- | --- | --- | --- |
| **TITLE** | | |  |
| Title | 1 | Identify the report as a systematic review. | Title (page 1). |
| **ABSTRACT** | | |  |
| Abstract | 2 | See the PRISMA 2020 for Abstracts checklist. | A completed PRISMA 2020 for Abstracts checklist is provided as Supplementary material. |
| **INTRODUCTION** | | |  |
| Rationale | 3 | Describe the rationale for the review in the context of existing knowledge. | Background (pages 4-7). |
| Objectives | 4 | Provide an explicit statement of the objective(s) or question(s) the review addresses. | Aim (page 8). |
| **METHODS** | | |  |
| Eligibility criteria | 5 | Specify the inclusion and exclusion criteria for the review and how studies were grouped for the syntheses. | Inclusion and exclusion criteria: Methods/ the Study Eligibility section (page 9). The synthesis of the studies: Methods/ Data Extraction and Data Synthesis (pages 11-12). |
| Information sources | 6 | Specify all databases, registers, websites, organisations, reference lists and other sources searched or consulted to identify studies. Specify the date when each source was last searched or consulted. | Methods/ Search Methods (page 9). Detailed search strategies for each database: Supplementary material. |
| Search strategy | 7 | Present the full search strategies for all databases, registers and websites, including any filters and limits used. | Methods/ the Search Methods (page 9). Detailed search strategies for each database: Supplementary material. |
| Selection process | 8 | Specify the methods used to decide whether a study met the inclusion criteria of the review, including how many reviewers screened each record and each report retrieved, whether they worked independently, and if applicable, details of automation tools used in the process. | The inclusion criteria: Methods/ Study Eligibility (page 9). Details of studies retrieved and included: Results (page 27), and the PRISMA flowchart on page 28. |
| Data collection process | 9 | Specify the methods used to collect data from reports, including how many reviewers collected data from each report, whether they worked independently, any processes for obtaining or confirming data from study investigators, and if applicable, details of automation tools used in the process. | Data extraction methods: Methods/ Data Extraction and Data Synthesis (pages 11-12). |
| Data items | 10a | List and define all outcomes for which data were sought. Specify whether all results that were compatible with each outcome domain in each study were sought (e.g. for all measures, time points, analyses), and if not, the methods used to decide which results to collect. | Methods/ Data Extraction and Data Synthesis (pages 11-12). |
|  | 10b | List and define all other variables for which data were sought (e.g. participant and intervention characteristics, funding sources). Describe any assumptions made about any missing or unclear information. | Methods/ Data Extraction and Data Synthesis (pages 11-12) and the Evidence Table 1 (pages 13-26). |
| Study risk of bias assessment | 11 | Specify the methods used to assess risk of bias in the included studies, including details of the tool(s) used, how many reviewers assessed each study and whether they worked independently, and if applicable, details of automation tools used in the process. | Methods/Quality Appraisal (page 10). |
| Effect measures | 12 | Specify for each outcome the effect measure(s) (e.g. risk ratio, mean difference) used in the synthesis or presentation of results. | Methods/ Data Extraction and Data Synthesis (page 12) and Results/ Description of the Included Studies (page 27-28). |
| Synthesis methods | 13a | Describe the processes used to decide which studies were eligible for each synthesis (e.g. tabulating the study intervention characteristics and comparing against the planned groups for each synthesis (item #5)). | Methods/ Study Eligibility (pages 9-10). Results/ Description of the Included Studies (pages 9-10) and Figure 1, the PRISMA flowchart. |
|  | 13b | Describe any methods required to prepare the data for presentation or synthesis, such as handling of missing summary statistics, or data conversions. | Methods/ Data Extraction and Data Synthesis (pages 11-12). |
|  | 13c | Describe any methods used to tabulate or visually display results of individual studies and syntheses. | Methods/ Data Extraction and Data Synthesis (pages 11-12) and Evidence Table 1. |
|  | 13d | Describe any methods used to synthesize results and provide a rationale for the choice(s). If meta-analysis was performed, describe the model(s), method(s) to identify the presence and extent of statistical heterogeneity, and software package(s) used. | Methods/ Data Extraction and Data Synthesis (pages 11-12). |
|  | 13e | Describe any methods used to explore possible causes of heterogeneity among study results (e.g. subgroup analysis, meta-regression). | Methods/ Data Extraction and Data Synthesis (pages 11-12) and Evidence Table 1. |
|  | 13f | Describe any sensitivity analyses conducted to assess robustness of the synthesized results. | No formal sensitivity analyses were conducted, as this review used a thematic synthesis approach. |
| Reporting bias assessment | 14 | Describe any methods used to assess risk of bias due to missing results in a synthesis (arising from reporting biases). | As this review did not conduct a meta-analysis, a formal assessment of reporting bias was not performed. However, a quality appraisal was conducted. Methods/ Quality Appraisal (Page 10). |
| Certainty assessment | 15 | Describe any methods used to assess certainty (or confidence) in the body of evidence for an outcome. | Methods/ Quality Appraisal (Page 10). |
| **RESULTS** | | |  |
| Study selection | 16a | Describe the results of the search and selection process, from the number of records identified in the search to the number of studies included in the review, ideally using a flow diagram. | Results/ Description of the Included Studies (27-28), as well as Figure 1, the PRISMA flow chart on page 28. |
|  | 16b | Cite studies that might appear to meet the inclusion criteria, but which were excluded, and explain why they were excluded. | The studies were cited in the results section. The reasons for excluding the studies: Results/ Description of the Included Studies (page 27), and Figure 1 (the PRISMA flow chart) on page 28. |
| Study characteristics | 17 | Cite each included study and present its characteristics. | The studies were cited in the Results and Evidence Table 1. |
| Risk of bias in studies | 18 | Present assessments of risk of bias for each included study. | Results/ Quality Appraisal (page 29), and the scores of the quality appraisal in the Evidence Table 1. |
| Results of individual studies | 19 | For all outcomes, present, for each study: (a) summary statistics for each group (where appropriate) and (b) an effect estimate and its precision (e.g. confidence/credible interval), ideally using structured tables or plots. | Because this review did not perform a meta-analysis, no pooled effect estimates were calculated. Instead, key findings from each study were summarized in the Evidence Table 1. |
| Results of syntheses | 20a | For each synthesis, briefly summarise the characteristics and risk of bias among contributing studies. | Results/ Evidence Table 1. |
|  | 20b | Present results of all statistical syntheses conducted. If meta-analysis was done, present for each the summary estimate and its precision (e.g. confidence/credible interval) and measures of statistical heterogeneity. If comparing groups, describe the direction of the effect. | No statistical synthesis or meta-analysis was performed. |
|  | 20c | Present results of all investigations of possible causes of heterogeneity among study results. | Methods/ Data Extraction and Data Synthesis (pages 9-10). |
|  | 20d | Present results of all sensitivity analyses conducted to assess the robustness of the synthesized results. | No statistical synthesis or meta-analysis was performed |
| Reporting biases | 21 | Present assessments of risk of bias due to missing results (arising from reporting biases) for each synthesis assessed. | No sensitivity analyses were conducted, as this review used a thematic synthesis approach. |
| Certainty of evidence | 22 | Present assessments of certainty (or confidence) in the body of evidence for each outcome assessed. | As no meta-analysis was performed, no formal sensitivity analyses were conducted. The robustness of the synthesized findings was assessed using a quality appraisal tool, Results/ Quality Appraisal (pages 12-13) and the scores of the quality appraisal in the Evidence Table 1 |
| **DISCUSSION** | | |  |
| Discussion | 23a | Provide a general interpretation of the results in the context of other evidence. | This point is addressed throughout the Results and Discussion sections. |
|  | 23b | Discuss any limitations of the evidence included in the review. | Discussion/ Limitations pages (41-42). |
|  | 23c | Discuss any limitations of the review processes used. | Discussion/ Limitations pages (41-42). |
|  | 23d | Discuss implications of the results for practice, policy, and future research. | Discussion/ implication and Recommendations (pages 42-45). |
| **OTHER INFORMATION** | | |  |
| Registration and protocol | 24a | Provide registration information for the review, including register name and registration number, or state that the review was not registered. | Methods/ Design (page 9). |
|  | 24b | Indicate where the review protocol can be accessed, or state that a protocol was not prepared. | Methods/ Design (page 9). |
|  | 24c | Describe and explain any amendments to information provided at registration or in the protocol. | Not applicable. |
| Support | 25 | Describe sources of financial or non-financial support for the review, and the role of the funders or sponsors in the review. | Declarations (page 46-47). |
| Competing interests | 26 | Declare any competing interests of review authors. | Declarations (page 46-47). |
| Availability of data, code and other materials | 27 | Report which of the following are publicly available and where they can be found: template data collection forms; data extracted from included studies; data used for all analyses; analytic code; any other materials used in the review. | Declarations (page 46-47). |

*From:*  Page MJ, McKenzie JE, Bossuyt PM, Boutron I, Hoffmann TC, Mulrow CD, et al. The PRISMA 2020 statement: an updated guideline for reporting systematic reviews. BMJ 2021;372:n71. doi: 10.1136/bmj.n71
